# A Relation Between Autism Traits and Gender Self-concept: Evidence from Explicit and Implicit Measures

**DOI:** 10.1007/s10803-019-04262-z

**Published:** 2019-10-24

**Authors:** Aimilia Kallitsounaki, David Williams

**Affiliations:** 1 School of Psychology, University of Kent, Canterbury, UK; 2 School of Psychology, Keynes College, University of Kent, Canterbury, CT2 7NP UK

**Keywords:** Autism spectrum disorder, ASD traits, Gender identity difficulties, Gender self-concept, Implicit Association Test

## Abstract

A link between autism spectrum disorder (ASD) and gender identity difficulties has been suggested. In this study, we found that, among adults from the general population (*N* = 101) ASD traits (measured using the Autism-spectrum Quotient) were associated negatively and significantly with the strength of both explicit gender self-concept (measured using the Personal Attributes Questionnaire) and implicit gender self-concept (measured using an Implicit Association Task). Further analyses showed that a subgroup with high/clinically significant ASD traits showed significantly weaker explicit and implicit gender self-concepts than a subgroup with low ASD traits. Results were similar in both males and females, although there was some evidence of a selective influence of ASD traits on implicit gender self-concept among females only.

**Electronic supplementary material:**

The online version of this article (10.1007/s10803-019-04262-z) contains supplementary material, which is available to authorized users.

Gender describes a constellation of traits, behaviours, and roles attributed to males and females within society (Wood and Eagly 2009). When individuals impute these cultural meanings of being male or female to themselves, then the process of formation and consolidation of a gender self-concept (or gender identity) begins (Wood and Eagly 2009, 2010, 2015). Traditionally, the study of gender self-concept in adults has relied on self-reports of the extent to which individuals believe their personality traits and interests conform to societal standards of masculinity and femininity. Research has shown that people attribute to themselves both feminine and masculine traits, with birth-assigned-males endorsing more masculine attributes as self-descriptive, and with birth-assigned-females endorsing more feminine attributes to themselves, on average (Bem 1974; Spence and Helmreich 1978).

Despite their extensive use in research, these self-report measures rely on accurate self-awareness of one’s own traits (which is unlikely to always be the case) and are considered susceptible to the effects of self-presentation (Devos et al. 2012; Nosek et al. 2005, 2007). That is, people may (consciously or nonconciously) attempt to camouflage or manipulate presentation of aspects of their selves and identities for various reasons. An alternative approach to use of verbal self-report measures of gender self-concept is to employ implicit measures that rely on some or other behavioural indicator of identity. Such, implicit measures are considered less prone than self-report measures to the adverse effects of self-presentation and have, therefore, been widely employed in research as an alternative to explicit measures of self and identity (e.g., Stieger et al. 2014; van Well et al. 2008). As Nosek et al. (2007) argue,

implicit cognition could reveal associative information that people were either unwilling or unable to report. In other words, implicit cognition could reveal traces of past experience that people might explicitly reject because it conflicts with values or beliefs, or might avoid revealing because the expression could have negative social consequences. Even more likely, implicit cognition can reveal information that is not available to introspective access even if people were motivated to retrieve and express it. (p. 266).

One of the most prominent of these measures is the Implicit Association Test (IAT; Greenwald et al. 1998). The IAT measures the strength of the automatic associations between concepts and attributes based on the assumption that strong links between concepts and attributes trigger faster behavioural reactions than concepts and attributes that are only weakly related with each other (Greenwald et al. 1998). The IAT requires respondents to identify and sort items into one of four categories, using two response keys, with each of the keys being assigned to two of the four categories. For example, in the gender self-concept IAT respondents sort words (e.g., I, They, Forceful, and Warm) that belong to one of the four following categories: Self/Other/Masculine/Feminine. Findings from the classic Greenwald and Farnham (2000) study using the gender self-concept IAT showed that birth-assigned-females tend to respond faster when the category of self shares the same response key as traditionally feminine attributes than when self shares response key with traditionally masculine attributes. In contrast, birth-assigned-males tend to show the opposite pattern (faster responses when the category of self is paired with masculine attributes than with feminine attributes).

The study of gender self-concept is particularly important when it comes to understanding of disorders that have been linked with gender identity difficulties, such as autism spectrum disorder (ASD). ASD is a neurodevelopmental disorder diagnosed on the basis of significant behavioural difficulties with social-communication and a restricted, repetitive pattern of interests and behaviour (American Psychiatric Association 2013). Williams et al. (1996) were the first who described the case of two boys diagnosed with ASD with co-occurring gender identity difficulties, expressed by cross-gender stereotyped interests and behaviours. Since then, a series of other cases studies followed, indicating the existence of a link between ASD and gender identity difficulties (e.g., Jacobs et al. 2014; Kraemer et al. 2005; Landén and Rasmussen 1997; Mukaddes 2002).

Indeed, de Vries et al. (2010) found that the prevalence of ASD among individuals with gender dysphoria (i.e., psychiatric disorder characterised by an incongruence between one’s own assigned gender at birth and their experienced/reported gender) was ten times greater than the population estimate of ASD. There has been also evidence about increased gender dysphoric traits among individuals with ASD (George and Stokes 2018) and an increased likelihood to express the wish to be the opposite gender (Janssen et al. 2016; May et al. 2017; Strang et al. 2014; van der Miesen et al. 2018) and to report atypical gender identities (e.g., Bejerot and Eriksson 2014; George and Stokes 2018).

Despite a suggestive link between ASD and gender identity difficulties, only two studies have examined whether individuals with ASD internalise attributes and roles that stereotypically define their biological sex to the same degree as neurotypical individuals do (Bejerot and Eriksson 2014; Stauder et al. 2011). Both studies used self-report measures of gender self-concept, and found that adults with ASD showed weaker conformity to masculine roles, traits, and interests than neurotypical adults. Interestingly, both studies found *non*-significant between groups differences in self-identification with feminine traits, although statistical power to detect even moderate group differences was relatively low in each study, which raises the possibility of a Type 2 error being made in each study.

In the current study, we attempted to address the issue of gender self-concept in ASD by taking an individual differences approach among a general population sample. ASD is now considered by most to be a dimensional disorder with autism traits being normally distributed in the general population (Constantino and Todd 2000, 2003; Ronald et al. 2006). As such, people from the general population will vary in the number of ASD traits they have from very few at one end of the spectrum to very high at the other end of the spectrum. Only at an arbitrary point toward the upper end of the distribution are individuals with a sufficiently high number of ASD traits given an official clinical diagnosis of ASD (Bolton et al. 1994; Goldberg et al. 2005; Le Couteur et al. 1996; Murphy et al. 2000; Pickles et al. 2000; Piven et al. 1997; Szatmari et al. 2008).

Therefore, we can learn important things about the nature and basis of gender identity in ASD by studying how gender self-concept varies according to the number of ASD traits manifested by people from the general population. Crucially, we included in the current study not only an explicit self-report measure of gender self-concept, but also a gender IAT as an implicit behavioural measure of the strength of participants’ identification with masculine and feminine traits, respectively. Inclusion of an implicit measure is crucial, because one possible interpretation of previous findings of a reduced identification with gender differentiated traits among people with ASD is that self-report measures are less valid than they are among neurotypical individuals. Given that aspects of self-awareness are considered by many researchers to be diminished in ASD (e.g., Carruthers 2009; Gopnik 1993; Williams 2010), it may be that ASD is associated with gender identification difficulties not because of a diminished implicit self-experience of gender feelings, but merely because those feelings are not accurately represented and reported. If that was the case, then ASD traits would be associated (negatively) with explicit self-report strength of gender self-concept but not with implicit, behavioural measures of gender self-concept.

In the current study, we measured ASD traits using the Autism-spectrum Quotient (AQ; Baron-Cohen et al. 2001) and the strength of the explicit gender self-concept using the Personal Attributes Questionnaire (PAQ; Spence and Helmreich 1978). Based on the existing preliminary findings about a weaker self-attribution of masculine traits among individuals with ASD (Bejerot and Eriksson 2014; Stauder et al. 2011) and the presence of gender identity difficulties also in birth-assigned-females with ASD (e.g., Lemaire et al. 2014), we predicted that the number of ASD traits would be negatively and significantly associated with the strength of the explicit gender self-concept. In other words, we expected that, as the number of ASD traits increased, the strength of explicit gender self-concept (i.e., the extent to which a person reports themselves to identify themselves masculine and feminine traits) would decrease.

Perhaps most importantly, however, we explored the relation between ASD traits and implicit gender self-concept for first time, employing the gender self-concept IAT described by Greenwald and Farnham (2000). Following the theory that ASD is associated with an underlying difficulty “identifying” with others (e.g., Hobson 2010; Hobson and Lee 1999; Tomasello 1999) we predicted that the strength of implicit gender self-concept would be also associated significantly with number of ASD traits, such that as ASD traits increase, so the strength of implicit gender self-concept will decrease.^1^

## Method

### Participants

One hundred and one adults (50 female) took part in the current online experiment. Their average age was 36.93 (SD = 10.11; range 22 to 70) years. Ninety-four percent of participants reported English as their first language and all were cisgender. Thirteen of the 101 participants had a formal diagnosis of autism, according to self-report. Participants were recruited through the Amazon’s online crowdsourcing platform MTurk and received compensation for their time. Informed consent was obtained from all individual participants included in the study. Ethical approval for this study was obtained from Kent School Psychology Research Ethics Committee.

### Materials and Procedure

#### Implicit Association Test

**Fig. 1:**
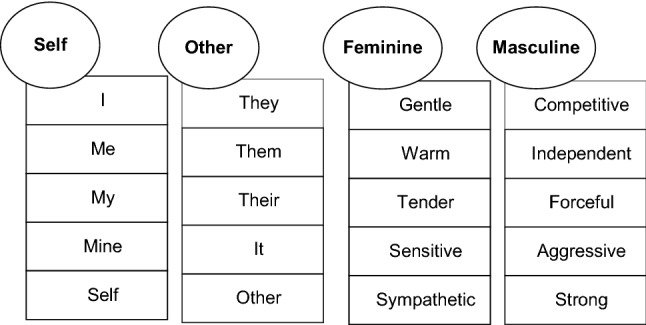
Contrast concepts and items presented in the gender self-concept Implicit Association Task

To measure implicit gender self-concept, we employed the IAT described by Greenwald and Farnham (2000). The task involved sorting words (see Fig. 1) that belonged to one of four categories (self/other/feminine/masculine), using one of two possible keys.

Words appeared on the middle of a computer screen in a random order and category labels were always presented in the upper right and left corners of the screen.

All stimuli were presented to participants before the beginning of the task, and they were instructed to study the items that belonged to each category for 25 s. In block 1, the “self” category label was presented in the upper left corner of the screen and the “other” category label in the upper right corner. Participants had to sort words that belonged either to the “self” or the “other” category, by pressing the “z” key of a keyboard for words related to “self” and the “m” key for words related to “other”. In block 2, categories referred to “feminine” and “masculine” attributes. The “feminine” category label was presented in the upper left corner and the “masculine” category in the upper right corner. Participants were instructed to press the “z” key for words belonged to “feminine” category and the “m” key for items belonged to “masculine” category.

In block 3, the four categories were presented combined (“self/feminine” labels: upper left corner; “other/masculine” labels: upper right corner) and participants practiced the sorting task, by pressing the key that was assigned to each category in the preceding two blocks (i.e., “z” key for items belonged to “self/feminine” categories and “m” key for items belonged to “other/masculine” categories). In block 4, they completed the first experimental condition of the sorting task. In block 5, the “self” category label was presented in the upper right corner and the “other” category label in the upper left corner, subsequently the assignment of the key for each category was reversed compared to the first block. Participants had to press “m” for items belonged to the “self” category and “z” for items belonged to the “other” category.

**Fig. 2:**
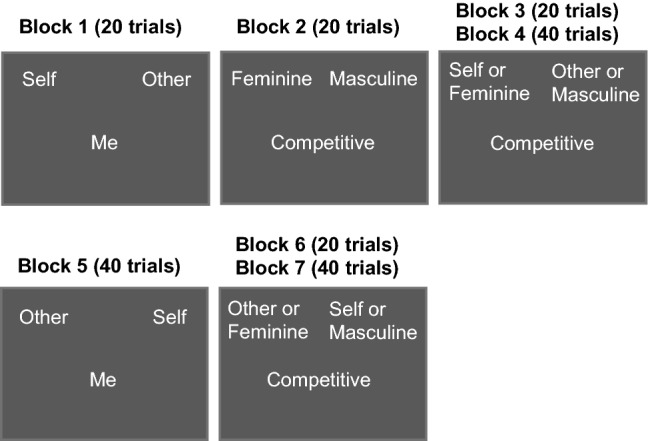
Illustration of the procedure and stimuli used in the gender self-concept Implicit Association Task

In block 6, they practiced the combined task using the switched key assignments. That is, participants were instructed to press the “z” key to categorise items that belonged either to the “other” or to the “feminine” category and the “m” key for words that belonged either to the “self” or “masculine” category. In the last block, they completed the second experimental condition of the combined sorting task. The order of the blocks was counterbalanced across participants. Figure 2 illustrates the procedure and the number of trials used in each of the seven blocks of the task.

The dependent measure for the IAT was the strength of the automatic associations calculated with the scoring algorithm recommended by Greenwald et al. (2003). That is, a standardised mean difference score (namely *D*) in response latencies between the two practical blocks (i.e., block 3 and block 6) and the two experimental blocks (i.e., bock 4 and block 7). The resulting *D* scores formulate a bipolar scale that ranges from masculinity to femininity. The faster the responses in the “self-masculine vs other-feminine” combined sorting condition, than in the “self-feminine vs other-masculine” condition, the stronger the implicit masculine gender self-concept. Whereas, the faster the responses in the “self-feminine vs other-masculine” combined sorting condition than in, the “self-masculine vs other-feminine” condition, the stronger the implicit feminine gender self-concept. However, in the current study, the key dependent variable on the IAT related to the strength of the implicit gender self-concept, regardless of whether the participant was male or female. Therefore, all *D* scores were transformed to positive values with higher scores denoting stronger implicit gender self-concept.

#### Personal Attributes Questionnaire (PAQ)

The PAQ (Spence and Helmreich 1978) is one of the most widely used self-report measures of explicit gender self-concept (Greenwald and Farnham 2000; Van Well et al. 2007; Ward et al. 2006). Participants are presented with 24 trait dimensions, with their endpoints being labelled with contradictory attributes (e.g., “Not at all aggressive–Very aggressive”) and they are asked to indicate the extent to which each item applies to them, using a 5-point scale. The questionnaire consists of an 8-item bipolar scale of gender self-concept that ranges from extreme femininity to extreme masculinity (F–M) and of two 8-item unipolar scales that measure masculinity (M) and femininity (F), separately. As such, three mean scores were calculated for each participant. High scores on the bipolar (F–M) indicate a masculine self-concept and low scores a feminine self-concept. The higher the score on the femininity (F) scale the stronger the feminine self-concept and the higher the score on the masculinity (M) scale the stronger the masculine self-concept for females and males, respectively. In terms of its psychometric properties, Spence and Helmreich (1978) reported that Cronbach’s alpha was .85, .82, and .78 for the masculinity, femininity, and femininity–masculinity scale, respectively. Moreover, scale scores clearly distinguish birth-assigned-males from birth-assigned-females (e.g., Helmreich et al. 1981) and the measure shows convergent validity (coefficients range from .52 to .84) with other self-report measures of gender self-concept (e.g., Bem Sex Role Inventory; Lubinski et al. 1983; Spence 1991).

#### Autism-Spectrum Quotient (AQ)

The AQ (Baron-Cohen et al. 2001) is a reliable and valid self-report measure of autism traits that has been widely used in research among clinical and non-clinical populations (e.g., Robertson and Simmons 2013; Williams et al. 2018a). Participants are asked to indicate their agreement with each of the 50 self-referential statements that the AQ comprises (e.g., “I find social situations easy”), using a four point Likert scale that ranges from “definitely agree” to “definitely disagree”. The total score ranges from zero to 50, with a value ≥ 26 indicating a clinically significant number of autism traits (Woodbury-Smith et al. 2005). The AQ shows acceptable test–retest reliability scores (e.g., *r* = .70; Baron-Cohen et al. 2001; *r* = .95; Broadbent et al. 2013) and convergent validity with the Social Responsiveness Scale, for example coefficients between .55 and .64 (Armstrong and Iarocci 2013; Ingersoll et al. 2011).

### Statistical Analysis

The focus of the current study was on the *strength* of gender self-concept, regardless of which direction the self-concept was in (i.e., masculine or feminine). Therefore, only the dimensional scales of PAQ and the transformed *D* scores of the IAT were included in the analyses described below. The bipolar scales of these measures were used only in an initial analysis to explore the association with untransformed *D* score, for comparison with the equivalent result reported by Greenwald and Farnham (2000). To examine the relation between ASD traits and the strength of the explicit/implicit gender self-concept, a series of zero-order correlations was conducted, with coefficients *r* being reported as measures of effect size (≥ .10 = small effect, ≥ .30 = moderate effect, ≥ .50 = large effect; Cohen 1992). Furthermore, a series of independent samples *t* tests was employed to examine differences in the explicit and implicit measures of gender self-concept between a low-AQ group and a high-AQ group. To categorise participants as having high or low number of self-reported ASD traits, we employed the widely used median split approach (e.g., Bayliss and Kritikos 2011; O’Keefe and Lindell 2013). That is, individuals who scored below the median score on the AQ (i.e. Mdn = 20.00) were assigned in the low AQ-group, whereas people who scored above the median were categorised in the high-AQ group. Cohen’s *d* values (≥ 0.20 = small effect, ≥ 0.50 = moderate effect, ≥ 0.80 = large effect; Cohen 1969) are reported as measures of effect size. A Chi Square test was also employed to explore whether there was a significant association between the group of participants (low-AQ/high-AQ) and gender. Cramer’s V was reported as measure of effect size and was interpreted as a coefficient *r* (McHugh 2013). To examine sex differences, a series of correlation analyses was conducted for birth-assigned-males and birth-assigned females separately and sex was used as a between-participants variable in a series of ANOVAs of the case–control data. Nonetheless, we should note here that sex differences were beyond the scope of the current study. Therefore, all were conducted post hoc and we did not make specific predictions about them.

An alpha level of .05 was used as the conventional criterion for statistical significance, unless a priori directional predictions were made. In those cases, values for one-tailed tests are reported. In addition to *p* values and effect sizes, we calculated another indicator that allowed us to make inferences based on our data (e.g., Dienes 2014; Rouder et al. 2009). Bayesian analyses are complementary to null hypothesis significance testing that is being used increasingly in ASD research (e.g., Nicholson et al. 2018; Williams et al. 2018a, 2018b). According to Jeffreys’s (1961) criteria, Bayes factors > 1 indicate increasing evidence for the alternative hypothesis over the null hypothesis (1–3 = anecdotal evidence; 3–10 = substantial evidence; 10–30 = strong evidence; 30–100 = very strong; values > 100 = decisive evidence). Whereas, scores < 1 indicate evidence for the null hypothesis over the alternative hypothesis (1–0.33 = anecdotal evidence; 0.33–0.10 = substantial evidence; 0.10–0.03 = strong evidence; 0.03–0.01 = very strong evidence; scores < 0.01 decisive evidence). When a priori directional predictions were made, BF_10_ values for one-tailed tests are reported. Bayesian analyses were performed using the statistical software package JASP 0.8.1.2 (JASP Team 2016).

In the current study, 13 participants reported possession of a formal diagnosis of ASD. Nonetheless, we did not exclude them from any analysis conducted in this study on the basis that ASD describes a spectrum disorder that ranges from people in the general population with low levels of autism traits to people who hold a clinical diagnosis of ASD (broad autism phenotype; e.g., Murphy et al. 2000; Pickles et al. 2000; Szatmari et al. 2008). However, in order to ensure that the significant effects we found were not inflated by their inclusion, we reconducted the main analyses including only non-ASD participants. Results did not change substantively (see Online Supplementary Material).

## Results

### Preliminary Analyses

A correlation analysis was conducted to examine the association between the explicit and the implicit measure of gender self-concept. The bipolar scale of PAQ was negatively and significantly associated with the untransformed *D* scores of the IAT, *r* = − .26, *p* = .009, BF_10_ = 3.55, showing that the more a person explicitly endorses masculine traits as self-descriptive, the more they implicitly/automatically associate their self-concept with masculine traits on the IAT. A Fisher’s *Z* test showed that the magnitude of the association was of equivalent size with that reported in the IAT original study by Greenwald and Farnham (2000), *Z* = − 0.36, *p* = .720.

The average AQ score across participants was 19.65 (SD = 7.31). The average score on PAQ Masculinity and PAQ Femininity scale was 2.43 (SD = 0.73) and 2.85 (SD = 0.72), respectively. Participants were accurate when sorting items on the IAT. The mean proportion of correct item categorisation (*M* = 85.94; SD = 13.90) in the critical blocks was above chance, *t*(100) = 62.12, *p* < .001, *d* = 6.18, BF_10_ = 6.99. Likewise, participants showed the expected effect on the IAT, producing a mean *D*-score (*M* = 0.39; SD = 0.32) that was significantly above zero, *t*(100) = 12.13, *p* < .001, *d* = 1.21, BF_10_ = 2.03.

### Association Analyses

A series of correlation analyses was conducted to explore the relation between ASD traits, on the one hand, and the explicit and implicit gender self-concept, on the other hand. In line with our predictions, AQ was negatively and significantly associated with both PAQ Femininity and PAQ Masculinity scale scores, *r* = − .43, *p* < .001, BF_10_ > 100 (one-tailed) and *r* = − .35, *p* < .001, BF_10_ > 100 (one-tailed), respectively. These results indicate that the higher the number of ASD traits manifested, the lower the explicitly reported strength of gender self-concept (for both feminine and masculine traits). Next, we examined the relation between AQ score and implicit gender self-concept (indexed by *D* score). As predicted, AQ was negatively and significantly associated with *D* score, *r* = − .25, *p* = .006, BF_10_ = 5.45 (one-tailed), indicating a negative association between the number of self-reported ASD traits and the strength of the implicit gender self-concept (high AQ score = low *D* score).

### Case–Control Analyses

Table 1 presents mean (*SD*) scores on PAQ Femininity and PAQ Masculinity scale and on the IAT among participants who scored above and below the median of the sample on the AQ.

**Table 1:** Means (SDs) and Inferential Statistics for Group Differences

	Group		Group differences			BF_10_^a^
	Low AQ	High AQ	*t*	*p*^a^	*d*	
	(*n *= 51)	(*n *= 50)				
IAT (*D*-score)	0.46 (0.38)	0.31 (0.23)	2.57	.006	0.51	7.21
PAQ Femininity	3.09 (0.55)	2.61 (0.80)	3.52	< .001	0.70	85.92
PAQ Masculinity	2.69 (0.64)	2.18 (0.74)	3.71	< .001	0.74	> 100

*IAT* Implicit Association Task, *PAQ* Personal Attributes Questionnaire

^a^Values for one-tailed tests are reported

A series of independent samples *t*-tests was conducted to examine differences between the low-AQ group and the high-AQ group in the explicit and implicit gender self-concept. Groups were equated for age and sex. The average age was 38.61 (SD = 9.02) in the low-AQ group and 35.22 (SD = 10.93) in the high-AQ group, a difference that was non-significant, *t*(94.85) = 1.70, *p* = .093, *d* = 0.38, BF_10_ = 0.75. The low-AQ group included 23 birth-assigned-males and 28 birth-assigned-females, whereas the high-AQ group included 28 birth-assigned-males and 22 birth-assigned-females. The difference between the high and low AQ groups in ratio of birth-assigned-males to birth-assigned-females was non-significant, χ^2^(1) = 1.20, *p* = .273, Cramer’s V = .11. As predicted, relative to participants in the low-AQ group, those in the high-AQ group had significantly lower scores on both PAQ scales, as well as significantly lower *D* scores on the IAT (see Table 1). When the AQ threshold of 26 (i.e., the clinical cut-off score) was used to split participants in a high-AQ group and in low-AQ group results remained essentially the same. The results from these analyses are reported in Online Supplementary Material.

### Exploratory Analyses Among Each Sex Separately

In a set of exploratory analyses, we checked whether there were any of the effects reported above differed significantly between birth-assigned males and birth-assigned females. Fisher’s *Z* tests were used to establish whether the size of correlations between variables differed significantly between sexes. Sex was also used as a between-participants variable in a series of ANOVAs of the case–control data. Only two of these analyses were significant (all other *p*s > .246).

First, there was a significant difference between males and females in the size of the association between *D* score on the gender IAT and total score on the AQ, *Z* = 2.49, *p* = .013. The association between AQ and *D* score was significant within birth-assigned-females, indicating that the higher the number of ASD traits, the lower the strength of the implicit gender self-concept, *r* = − .41, *p* = .003, BF_10_ = 11.30. However, this association was non-significant among birth-assigned-males, *r* = .08, *p* = .584, BF_10_ = 0.20.

Second, in a 2 (sex: birth-assigned-male/birth-assigned-female) × 2 (AQ subgroup: High-AQ/low-AQ) ANOVA on the gender IAT data, there was a significant group × sex interaction, *F*(1,97) = 11.02, *p* = .001, $\eta_{p}^{2}$ = .10. The IAT *D* score among birth-assigned-females in the low AQ subgroup (*M* = 0.65, SD = 0.40) was significantly higher than among birth-assigned-females in the high AQ subgroup (*M* = 0.33, SD = 0.26), *t*(46.73) = 3.44, *p* = .001, *d* = 0.96, BF_10_ = 17.73. This indicates that females with a high number of ASD traits had a weaker gender self-concept than females who had a low number of ASD traits. In contrast, the IAT *D* score among birth-assigned-males in the low AQ subgroup (*M* = 0.24, SD = 0.18) was non-significantly different from the IAT *D* score among birth-assigned-males in the high AQ subgroup (*M* = 0.29, SD = 0.20), *t*(49) = − 0.90, *p* = .379, *d* = 0.25, BF_10_ = 0.39.

## Discussion

The first notable set of findings of the current study was about the link between ASD traits and the explicit gender self-concept. As predicted, AQ score was significantly negatively associated with both Femininity and Masculinity scale scores of PAQ, indicating that as the number of self-reported ASD traits increases the strength of the explicit gender self-concept decreases. The associations were moderate in magnitude and Bayesian analyses consistently suggested that the data supported the alternative hypothesis. This is the first study, to our knowledge, to show a link between explicit gender self-concept and number of ASD traits among members of the general population. We also addressed this issue by dividing our sample into a high AQ group and a low AQ group. In keeping with our predictions, the results complemented the findings from the correlation analyses. That is, relative to the group of participants with low ASD traits, the group with high ASD traits scored significantly lower on both scales of PAQ. These results indicate that people with high ASD traits explicitly/consciously identify themselves with less strong masculine and feminine attributes relative to individuals with low ASD traits; they have weaker gender self-concepts, on average. Effects were moderate and Bayesian analyses again suggested that the data supported the alternative hypotheses.

These results are in keeping with previous findings that indicated people with a diagnosis of ASD tend to report a weaker masculine self-concept compared than do neurotypical individuals. This is important and increases our confidence in the reliability of previous findings linking ASD and weak masculine self-concept (Bejerot and Eriksson 2014; Stauder et al. 2011). Given that only two previous studies have explored this link, replication of earlier reported effects, but in the general population is notable and should make a valuable contribution to the field. More than this, however, the current study provides the first evidence that ASD is also linked to a diminished *feminine* gender identity/self-concept. The studies by Bejerot and Eriksson (2014) and Stauder et al. (2011) found that ASD and control samples self-reported equally strong identification with feminine traits, which led the authors of those studies to conclude that ASD was linked specifically with a reduced identification with masculine traits. While such a conclusion was reasonable on the basis of their findings, alternative explanations are possible. For example, given that Stauder et al.’s (2011) study had statistical power of only .26 to detect a predicted moderately-sized difference between birth-assigned-females with ASD (*n* = 9) and birth-assigned-females without ASD (*n* = 9) in Gender Feminine scale, it is possible that a Type 2 error was made. Also, the Bem Sex Role Inventory, used by Bejerot and Eriksson (2014), includes items that are significantly more desirable for one sex than the other (Hoffman 2001) and so could have confounded results in that study (e.g., if neurotypical birth-assigned-males failed to report feminine characteristics because of social undesirability).

Importantly, the current study included a self-report measure of gender self-concept that did not bias participants against self-endorsement of feminine traits, as it includes psychological traits that are equally desirable for both sexes (Hoffman 2001). Moreover, Bayesian analyses were conducted to provide a more complete picture of the results than available in previous studies of the link between gender self-concept and ASD. Given this, findings suggest that people with high ASD traits do report a less strong feminine self-concept compared to people with low ASD traits.

Arguably more notable, is the second set of findings from the current study, regarding the relation between number of ASD traits and implicit gender self-concept. To our knowledge, the current study is the first to investigate the potential link between ASD and gender self-concept at a level deeper than that tapped by self-report. In keeping with our predictions, we found that the strength of the implicit gender self-concept (indexed by *D*-score) was significantly negatively associated with number of self-reported ASD traits, indicating that as the number of reported ASD traits increased the strength of the implicit gender self-concept decreased. The size of the association was relatively modest (small-to-medium), but it was highly statistically significant and Bayesian analysis suggested that the data supported the alternative hypothesis. Complementary to this, relative to the group of participants with low ASD traits, the group with high ASD traits scored significantly lower (*D*-score) on the IAT, indicating that among people with high ASD traits the automatic identification of *self* with either masculine or feminine attributes was weaker relative to individuals with low ASD traits. Again, group differences were moderate-to-large in statistical magnitude and associated with Bayes factors that strongly favoured the alternative hypothesis. These results suggest that high ASD traits in the general population might signify an implicit/unconscious gender self-concept that is neither strongly masculine nor strongly feminine, among individuals who otherwise report themselves to be cisgender.

Importantly, the significant association we found between the implicit and the explicit measure of gender self-concept was equivalent in size to the one reported in the seminal study by IAT original study by Greenwald and Farnham (2000) (*r* = − .26 in our study and *r *= .20 in their study). Moreover, these associations are of an order of magnitude suggested by Hofmann et al. (2005) to indicate consistency between implicit and explicit measures. This is important, because it provides evidence that the two measures are tapping overlapping sets of representations about one’s gender, rather than tapping entirely different constructs. Of course, it should be noted that some divergence between implicit and explicit measures is always expected, given that “direct ratings are farther downstream in the processes of judgement and thus subject to more deliberation than the more spontaneous, automatic associations tapped by indirect measures” (Wood and Eagly 2009, p. 112). As a result, direct measures can be prone to the influence of social desirability.

A note of caution is also needed, when interpreting results from the gender IAT. In a series of post hoc analyses, we observed that the explicit effects (using the PAQ) observed in the whole sample of 101 participants held in *both* birth-assigned males (*n* = 51) and birth-assigned females (*n* = 50). That is, in the association analyses, the strength of explicit gender self-concept was negatively associated with number of ASD traits in both males and females. Likewise, in the case–control analyses, those with high ASD traits had a weaker gender self-concept than those with low ASD traits, regardless of whether they were male or female. However, results appeared to be not so straightforward when considering the effects of ASD traits on *implicit* gender self-concept. Here, in the association analyses, number of ASD traits was negatively associated with strength of implicit gender self-concept in birth-assigned females only. Likewise, in the case–control analyses, only females with high ASD traits had a weaker gender self-concept than females with low ASD traits (the same analyses in males revealed no between-group differences). What should be made of these apparent sex differences in the relation between ASD and implicit gender self-concept? On the one hand, the existence of gender differences might be treated with some scepticism, given that the analyses were entirely post hoc and between-sex differences were not predicted. On the other hand, sex differences were reported by Nobili et al. (2018) in their study of the association between ASD traits and transgender status. Nobili et al. (2018) found that birth-assigned-females in a transgender group reported significantly more ASD traits relative to birth-assigned-females in a cisgender group. No differences between gender groups in number of ASD traits were seen among birth-assigned-males in their study, however. As such, our results could be considered in line with these findings. Nevertheless, gender self-concept is different from transgender identity and therefore future studies need to examine sex differences in gender self-concept further before strong conclusions can be drawn.

Overall, the results from the explicit and implicit measures of gender self-concept imply that people with high ASD traits (females, at least) have a weaker propensity to identify with, and incorporate into their self-concept gender differentiated traits. As levels of ASD increase, so too may be susceptible to gender identity difficulties. If this interpretation is correct, then this could explain gender non-conforming feelings and gender identity difficulties in ASD (Glidden et al. 2016; Øien et al. 2018; Van Der Miesen et al. 2016).

Given that several researchers have argued (and provided evidence to show) that ASD is characterised by a diminished general tendency to identify with, or represent, the perspectives and attitudes of others (e.g., Baron-Cohen and Wheelwright 2004; Hobson and Lee 1999; Hobson et al. 2006; Tomasello 1999), it could be that gender-related attitudes are not internalised and incorporated into the self-concept of children with ASD in the same manner, or to the same depth, as among neurotypical children. In addition, difficulties representing others’ perspectives also contribute to reduced experiences of self-conscious emotion in children with ASD (e.g., Hobson et al. 2006). Whereas a neurotypical child might feel self-conscious or embarrassed at others’ reactions to their gender incongruent behaviour and thus seek to conform to gender-typical norms, a gender-incongruent child with ASD would likely be much less moved to change by the attitudes of others. As a result, with diminished pressure for conformity, gender-related attributes and roles might not easily become incorporated into the self-concept of children with ASD (e.g., Bargiela et al. 2016). This is important given, the dearth of evidence about a mechanism that could explain the link between ASD and gender identity difficulties (Glidden et al. 2016), but also because it denotes the continuous nature of gender and the influence of society upon the formation of the so-called binary gender identity (e.g., Ehrensaft 2018; Turban and van Schalkwyk 2018).

Moreover, this idea fits with some first person accounts by autistic people and about how they experience gender. As summarised by Davidson and Tamas (2016, p. 61), “not only does gender not constitute the definitive core of autistic experience, but for many, gender is barely present at all”. Our study, suggests that this claim does not reflect difficulties with self-awareness (in females, at least). Rather, among people with high ASD traits there seems to be a match between their internal/nonconscious experience of gender self-concept and their explicit expression of this facet of self. The extent to which there is also the same level of consistency between the implicit and the explicit experience of gender self-concept among people with a diagnosis of ASD is still debatable, until it is directly explored.

In conclusion, the current study suggests that people with high ASD traits have a weaker inclination not only to identify with gender differentiated traits, but also (females, at least) to incorporate these into their self-concept. Given that such a difficulty could provide an explanation for the high co-occurrence of gender identity difficulties in ASD, the current study makes an important contribution to our still limited understanding of this phenomenon.

## Electronic supplementary material

Below is the link to the electronic supplementary material.

Supplementary material 1 (DOCX 18 kb)

## Footnotes

1 The authors of the current paper had different predictions regarding the relation between ASD traits and the strength of the implicit gender self-concept. The first author (i.e., AK) predicted that the number of ASD traits would be negatively and significantly associated with the strength of the implicit gender self-concept, such that, as the number of ASD traits increased, the strength of the implicit gender self-concept would decrease. Whereas, the second author (i.e., DW) predicted a non-significant association.
