# Supplementary material for: A Relation Between Autism Traits and Gender Self-concept: Evidence from Explicit and Implicit Measures
Source: J Autism Dev Disord. 2019 Oct 24;50(2):429–39. doi: 10.1007/s10803-019-04262-z (PMC6994450; doi:10.1007/s10803-019-04262-z)
Supplement: Supplementary file 1 — Supplementary material 1 (DOCX 18 kb) [file 10803_2019_4262_MOESM1_ESM.docx]

**Supplementary Material**

**1. ASD participants excluded**

**Bivariate correlations**

When participants who reported possession of a formal diagnosis of ASD (*n* = 13) were excluded from the analysis results did not change substantively. AQ remained correlated negatively and significantly with PAQ Femininity and PAQ Masculinity scale scores, *r* = -.44, *p* < .001, BF_10_ > 100 (one-tailed) and *r* = -.35, *p* < .001, BF_10_ = 75.04 (one-tailed), respectively. Moreover, the correlation between AQ and *D* scores remained negative and significant, *r* = -.23, *p* = .014, BF_10_ = 2.78 (one-tailed).

**Case-control analyses**

We also examined differences between a low-AQ group and a high-AQ group in the size of explicit and implicit gender self-concept. Participants who scored below the median score on the AQ (i.e., *Mdn* = 19.00) were assigned in the low AQ-group (*n* = 46), whereas participants who scored above the median were categorised in the high-AQ group (*n* = 42). The average femininity score was 3.10 (*SD* = 0.57) in the low-AQ group and 2.63 (*SD* = 0.83) in the high-AQ group, a difference that remained significant, *t*(86) = 3.10, *p* = .002, *d* = 0.66, BF_10_ = 26.25 (one-tailed). The average masculinity score was 2.72 (*SD* = 0.64) in the low-AQ group and 2.18 (*SD* = 0.80) in the high-AQ group, a difference that remained significant, *t*(86) = 3.49, *p* = .001, *d* = 0.74, BF_10_ = 76.45 (one-tailed). The average *D* score was 0.48 (*SD* = 0.38) in the low-AQ group and 0.31 (*SD* = 0.23) in the high-AQ group, a difference that remained significant, *t*(75.46) = 2.62, *p* = .006, *d* = 0.55, BF_10_ = 7.54 (one-tailed).

**2. Alternative way of splitting participants: AQ clinical cut-off score**

When we used the AQ threshold (i.e., the clinical cut-off score) of 26 to split participants in a high-AQ group (*n* = 21) and in a low-AQ group (*n* = 80), results remained essentially the same. The between-group difference in PAQ Femininity scale score remained significant, with the high-AQ group (*M* = 2.29; *SD* = 0.70) scoring lower than the low-AQ group (*M* = 3.00; *SD* = 0.66), *t*(99) = 4.33, *p* < .001, *d* = 1.04, BF_10_ > 100 (one-tailed). Likewise, people in the high-AQ group (*M* = 2.09; *SD* = 0.66) scored significantly lower in PAQ Masculinity scale than people in the low AQ group (*M* = 2.52; *SD* = 0.73), *t*(99) = 2.47, *p* = .008, *d* = 0.63, BF_10_ = 6.48 (one-tailed). In terms of the implicit measure of gender self-concept, the average *D* score (*M* = 0.29; *SD* = 0.26) in the high-AQ group was lower, relative to the average *D* score in the low-AQ group (*M* = 0.41; *SD* = 0.33) and that difference was marginally significant, *t*(99) = 1.56, *p* = .061, *d* = 0.41, BF_10_ = 1.31 (one-tailed).
